# Correction: Development of electrochemical sensors for quick detection of environmental (soil and water) NPK ions

**DOI:** 10.1039/d4ra90036g

**Published:** 2024-04-10

**Authors:** M. I. Hossain, M. A. Khaleque, M. R. Ali, M. S. Bacchu, M. S. Hossain, S. M. F. Shahed, Mohamed Aly Saad Aly, Md. Z. H. Khan

**Affiliations:** a Laboratory of Nano-Bio and Advanced Materials Engineering (NAME), Jashore University of Science and Technology Jashore 740S Bangladesh zaved.khan@yahoo.com; b Department of Chemical Engineering, Jashore University of Science and Technology (JUST) Jashore 740S Bangladesh; c Department of Chemistry, Graduate School of Science, Tohohi University Aramaki-Aza-Aoba, Aoba-Ku Sendai 980-8578 Japan; d Department of Electrical and Computer Engineering at Georgia Tech Shenzhen Institute (GTSI), Tianjin University Shenzhen Guangdong 5ISO52 China mohamed.alysaadaly@ece.gatech.edu

## Abstract

Correction for ‘Development of electrochemical sensors for quick detection of environmental (soil and water) NPK ions’ by M. I. Hossain *et al.*, *RSC Adv.*, 2024, **14**, 9137–9158, https://doi.org/10.1039/D4RA00034J.

The authors regret that the contribution of one of the authors (M. Aly Saad Aly) was incorrectly given. The corrected author contributions are as shown here.

The Royal Society of Chemistry apologises for these errors and any consequent inconvenience to authors and readers.

## Supplementary Material

